# A Unified Framework for Motion Estimation and Online Camera Orientation Calibration Using a Ground-View Event Camera

**DOI:** 10.3390/s26196032

**Published:** 2026-09-23

**Authors:** Junzhe Su, Masahiro Hirano, Yuji Yamakawa

**Affiliations:** The Institute of Industrial Science, The University of Tokyo, Meguro-ku, Tokyo 153-8505, Japan; mhirano@iis.u-tokyo.ac.jp (M.H.); y-ymkw@iis.u-tokyo.ac.jp (Y.Y.)

**Keywords:** unified framework, ground-view event camera, motion estimation, online camera orientation calibration, ground vehicles

## Abstract

Accurate motion estimation and online camera orientation calibration are important for onboard vision systems on ground vehicles. Ground-view sensing is attractive for these tasks because the ground is typically static, close to the vehicle, and continuously observable, but fast ground-relative motion requires high-temporal-resolution sensing. This paper presents a unified framework that uses a ground-view event camera to estimate ground-view camera motion and support online orientation calibration of a target vehicle-mounted camera. The event camera provides high temporal resolution with sparse measurements, making it suitable for fast ground-view sensing. The framework couples online event-based ground-view motion estimation with an improved calibration process, allowing the estimated motion to support calibration and be further refined during calibration. Specifically, ground-view motion is first estimated online from short event windows using a spiking neural network, providing direct short-term motion information from the event stream. The estimated motion is then integrated with target-camera motion and geometric constraints to estimate the target-camera orientation while further refining the ground-view motion. Experiments on real-vehicle data show accurate ground-view motion estimation, improved rotational components through motion refinement, and better target-camera orientation calibration, especially in yaw, while improvements in pitch and roll are less pronounced. These results demonstrate the effectiveness of integrating event-camera-based ground-view motion estimation and online target-camera orientation calibration for ground vehicles.

## 1. Introduction

Ground vehicles increasingly rely on robust environment perception for safe and efficient operation. Accurate spatial understanding of surrounding scenes is essential for tasks such as obstacle avoidance, navigation, and decision making, and also benefits localization and scene interpretation in complex driving environments. Among different sensing modalities, cameras are particularly important because they provide rich visual information at low cost. They support a wide range of perception functions, including obstacle detection, lane recognition, ego-motion estimation, and 3D scene understanding, and therefore have become indispensable in modern ground-vehicle perception systems.

Motion estimation is a key function of camera-based perception and is fundamental to the operation of ground vehicles [1]. Accurate motion estimation ensures stable navigation, enhances safety through precise braking and acceleration control, and improves motion planning in complex and dynamic environments [2]. Furthermore, it contributes to dynamic scene understanding by distinguishing the platform’s ego motion from independently moving objects, thereby facilitating predictive modeling of environmental dynamics and proactive collision avoidance. Compared with wheel encoders, camera-based methods are less affected by wheel slip and terrain irregularities [3]; compared with Inertial Measurement Unit (IMU)- or Global Positioning System (GPS)-based approaches, they can provide useful motion cues without cumulative drift or signal blockage issues in visually observable environments. These properties make camera-based motion estimation an important component of robust environment perception for ground vehicles.

Accurate camera extrinsic calibration is essential for fully exploiting camera-based perception in ground vehicles. In motion estimation, the estimated motion must be transformed from the camera frame to the vehicle frame, so calibration errors can directly affect downstream tasks such as localization, control, and vehicle-state estimation [4]. Precise extrinsic calibration is also important for other vision-based functions, including obstacle perception, scene reconstruction, and multi-sensor fusion [5]. In long-term operation, however, extrinsic parameters may gradually change due to vibration, thermal effects, mechanical impacts, or hardware modifications [6,7,8]. Although offline calibration with fiducial patterns such as checkerboards [9] can achieve high accuracy under controlled conditions, it typically requires expert intervention and carefully prepared environments, such as appropriate illumination, flat surfaces, and sometimes even dedicated calibration facilities. As a result, offline calibration is well suited to initial factory setup or laboratory calibration, where such conditions can be ensured. However, for repeated recalibration during practical deployment—especially in long-term operation or large-scale maintenance—it becomes considerably less convenient and often impractical. By contrast, online calibration estimates extrinsic parameters using information acquired during regular operation, reducing the need for manual intervention and enabling recalibration after disturbances or hardware maintenance. Online calibration can also relax the precision requirements of initial sensor installation, thereby simplifying system integration in real-world applications [10]. For ground vehicles operating over long periods and under changing conditions, it is therefore a more practical and scalable solution for maintaining geometric consistency in vision-based perception systems. In the context of this paper, online calibration denotes this in-operation setting, in which information acquired during regular vehicle operation is used without dedicated calibration targets or facilities; it does not imply real-time execution of the complete framework.

Despite the importance of camera-based motion estimation and online calibration, both remain challenging in practical ground-vehicle perception systems. Most existing visual systems mainly rely on forward- or side-facing cameras. For motion estimation, such outward-facing views often assume that sufficient static scene structure is consistently observable across consecutive frames. In real-world environments, however, this assumption is frequently violated by dynamic objects such as surrounding vehicles, pedestrians, or cyclists, which can corrupt visual motion cues and degrade estimation accuracy [11]. In addition, in visually sparse scenes such as highways, tunnels, corridors, or parking areas, the lack of distinctive features further limits the reliability of camera-based motion estimation. Online camera extrinsic calibration also faces practical limitations. Many existing methods [6,7,8,12,13,14,15,16] rely on artificial features such as lane markings or line structures, and their performance is therefore highly dependent on the availability and quality of such features. When these cues are weak, noisy, or partially absent, calibration accuracy can deteriorate significantly. Some studies [17,18,19] have attempted to improve robustness through multi-sensor fusion with IMUs, wheel encoders, or other modalities; however, this introduces additional challenges such as synchronization errors, sensor drift, and wheel-slip-related inaccuracies.

Given the above challenges in both camera-based motion estimation and online extrinsic calibration, ground-view cameras provide a promising complementary sensing modality for ground vehicles. Compared with surrounding objects that may move or become occluded, the ground surface is usually static and continuously observable, offering a stable source of visual and geometric information. In addition, because ground-view cameras are oriented downward, they are less likely to capture identifiable human features or other sensitive scene content, which is also advantageous from a privacy-preserving perspective [20]. For motion estimation, local textures and patterns on the ground surface can serve as useful cues for ego-motion inference, even when outward-facing cameras are affected by occlusion or poor feature visibility. These cues therefore provide an additional source of motion information that can complement conventional outward-facing perception. For online camera extrinsic calibration, the geometric consistency of the ground plane can be exploited to estimate camera orientation relative to the vehicle frame. Since ground vehicles typically undergo planar motion, the spatial relationship between the camera and the ground provides a reliable basis for continuous calibration [21]. By exploiting ground-related geometric constraints, the calibration can be carried out in a self-contained manner without requiring artificial patterns, visibility of the vehicle itself, or additional sensing modalities. This makes ground-view cameras well suited to robust and scalable maintenance of extrinsic accuracy during long-term operation of ground vehicles.

Although ground-view cameras offer useful complementary information, they also introduce additional challenges. Because they are mounted close to the ground, they are more susceptible to motion blur during fast motion or abrupt maneuvers. In addition, the overlap between consecutive observations can become limited, especially under rapid motion or when narrow-angle lenses are used, which makes it more difficult to establish reliable visual correspondences for motion estimation and calibration. These issues make high-temporal-resolution visual sensing particularly important in ground-view applications. In this context, event cameras are well suited to such scenarios because they asynchronously report brightness changes at individual pixels instead of capturing full image frames at fixed intervals [22]. Owing to this sensing mechanism, they provide extremely high temporal resolution, high dynamic range, and virtually no motion blur, while maintaining a sparse and efficient data representation. These properties make event cameras particularly suitable for fast ground-view sensing in both motion estimation and online calibration.

Motivated by the above observations, this paper investigates whether a single ground-view event camera can support both motion estimation and online camera orientation calibration within a unified framework. In this framework, the calibration task estimates the rotational component of the target-camera extrinsic parameters, while the translational component remains fixed at its initial value. Building on our previous studies on ground-view event-based motion estimation [23] and online calibration aided by a ground-view camera [21], the proposed framework processes the event stream captured by the ground-view event camera with a motion estimation algorithm to estimate the motion of the ground-view camera itself. In parallel, images from the target camera are used to estimate the motion of the target camera. These two types of motion estimates are then integrated into the calibration procedure used in the proposed framework to estimate the orientation of the target camera relative to the ground vehicle. To make better use of the ground-view motion estimate during calibration, the online calibration procedure is further improved so that the estimated motion of the ground-view camera can be refined together with the calibration parameters. This refinement improves the quality of the ground-view motion estimate and also benefits the final calibration accuracy.

The main contributions of this paper can be summarized as follows:

- We present a unified framework in which a single ground-view event camera supports both motion estimation and online camera orientation calibration for ground vehicles.
- Unlike our previous study [21], which used high-speed frame-based ground-view motion estimation, this paper integrates a ground-view event camera and a Spiking Neural Network (SNN)-based motion estimation method [23] into the unified framework. This enables metrically scaled motion estimation, improves flexibility under small camera-to-ground height variations, and, together with the event camera’s high temporal resolution, supports fast ground-view sensing.
- We improve our previous online calibration method so that the estimated motion of the ground-view camera can be further refined during calibration. This improvement enhances both the ground-view motion estimate and the final calibration accuracy. It also enables the framework to provide an accurate ground-view motion estimate with absolute scale, in contrast to typical monocular motion estimation methods whose outputs do not directly contain scale.

## 2. Problem Formulation and Method

The proposed framework uses a target camera and a ground-view event camera as inputs and addresses two interrelated tasks: online ground-view camera motion estimation and online target-camera orientation calibration. The image sequence captured by the target camera is used to obtain the target-camera motion estimate, while the event stream captured by the ground-view event camera is used to estimate the ground-view camera motion. These two motion estimates are then jointly used in the online target-camera orientation calibration process, through which the orientation of the target camera relative to the vehicle is estimated while the motion of the ground-view camera is further refined.

Accordingly, the framework outputs the calibrated target-camera orientation together with two motion-related quantities: the online ground-view camera motion estimate, which is the direct output of the motion-estimation branch, and the refined ground-view camera motion, which is obtained after the calibration process and reflects the further optimization of the estimated motion. The problem is formulated below using the geometric relationships among the two cameras, the ground plane, and the vehicle, after which the complete processing framework and its individual modules are described.

### 2.1. Problem Formulation

The proposed framework considers two cameras with different roles: a ground-view event camera, denoted as $camera_{i}$, whose event stream is used for motion estimation, and a target camera, denoted as $camera_{a}$, whose extrinsic orientation relative to the vehicle is to be calibrated. As shown in Figure 1, four coordinate systems are introduced: $C_{i}$ for $camera_{i}$, $C_{a}$ for $camera_{a}$, $C_{g}$ for the projection of $C_{i}$ onto the ground plane, and $C_{v}$ for the vehicle. In $C_{v}$, the *z*-axis points forward, the *x*-axis lies on the plane parallel to the ground and is perpendicular to the *z*-axis, and the origin is located at the midpoint of the rear-wheel axis on the ground.

Among the transformations shown in Figure 1, $T_{va}$ denotes the extrinsic parameters of $camera_{a}$ relative to the vehicle, and is decomposed into a rotation part $R_{va}$ and a translation part $t_{va}$. Since the calibration task in this study focuses on the orientation of $camera_{a}$, only $R_{va}$ is estimated, while $t_{va}$ is kept fixed at its initial value.

For the motion-estimation component, the quantity of interest is the motion of $C_{g}$, which represents the ground-parallel motion associated with $camera_{i}$. Let $T_{gi}$ denote the pose of $camera_{i}$ relative to $C_{g}$, and let $\Delta T_{g}^{k,k+1}$ denote the motion of $C_{g}$ from time *k* to time k+1. This motion consists of translation parallel to the ground plane and rotation about the ground normal, following the planar-motion assumption of the ground vehicle. Events captured by $camera_{i}$ within fixed-length temporal windows are converted into the input of the SNN, which estimates $\Delta T_{g}^{k,k+1}$.

Within the proposed unified framework, the estimated motion of $C_{g}$ has two roles: it is the output of the motion-estimation component, and it is also incorporated, together with the motion estimated from $camera_{a}$, into the subsequent online calibration procedure.

With the above definitions, the problem considered in this study can be stated as follows. Given the synchronized event stream from the ground-view event camera and the image sequence from the target camera, the objective is first to estimate the ground-parallel motion $\Delta T_{g}^{k,k+1}$ of the ground-view camera and then to estimate the target-camera orientation $R_{va}$ relative to the vehicle using this motion together with the target-camera motion. During the calibration process, $\Delta T_{g}^{k,k+1}$ is further refined, while the target-camera translation $t_{va}$ remains fixed at its initial value. Thus, the two estimation tasks are coupled: the online ground-view motion estimate supports target-camera orientation calibration, and the calibration process in turn improves the ground-view motion estimate.

### 2.2. Method

The overall framework of the proposed system is illustrated in Figure 2. As shown in the figure, the framework consists mainly of SNN-based ground-view motion estimation and online target-camera orientation calibration. The target-camera images are processed by a visual odometry algorithm (e.g., ORB-SLAM2 [24]) to obtain the target-camera motion estimate. In parallel, the event data captured by the ground-view event camera are fed into the SNN-based motion estimation module to produce the online ground-view camera motion estimate. These two motion estimates are incorporated into the calibration procedure based on the extrinsic-parameter invariance constraint and the nonholonomic constraint.

Since the event camera operates asynchronously, whereas the target camera acquires images at discrete time instants, temporal alignment between the two sensing modalities is required. To this end, each image acquisition event of the target camera is used as a synchronization signal and recorded in the event stream of the ground-view event camera through a special marker. In the present implementation, the target camera operates at 50 Hz; therefore, the events between two successive synchronization markers correspond to one 20 ms target-camera frame interval and are partitioned by timestamp into ten contiguous and non-overlapping 2 ms windows. The incremental ground-view motions estimated from these windows are then composed to obtain the ground-view motion over the same target-camera frame interval. The resulting ground-view motion estimate is temporally aligned with the target-camera motion estimate and can therefore be jointly used in the subsequent calibration process.

The ground-view motion-estimation and target-camera calibration components described above build on our previous studies on ground-view event-based motion estimation [23] and online calibration aided by a ground-view camera [21], respectively. The proposed unified framework advances these previous studies in two main respects. First, the high-speed frame-based ground-view motion estimation used in [21] is replaced by the ground-view event camera and SNN-based motion estimator developed in [23]. This integration exploits the high temporal resolution and sparse representation of event cameras for fast ground-view sensing, provides online metrically scaled motion estimates from short event windows, and improves flexibility under small camera-to-ground height variations. The resulting motion estimates serve both as direct outputs of the framework and as inputs to the calibration process. Second, we introduce a decoupled two-sub-step optimization strategy into the second step of our previous calibration method. Unlike the original coupled optimization, the proposed strategy first jointly refines the rotational relationship $R_{ga}$ and the rotational component of the ground-view motion $\Delta R_{g}^{k,k+1}$ under a rotation-only constraint, thereby reducing the influence of translation errors in $T_{ga}$. It then estimates the scaled rotation term $R_{ga}^{s}$ using the refined rotational alignment. This strategy improves the rotational component of the ground-view motion and benefits target-camera orientation calibration. The following subsections describe the two modules and the corresponding extensions in detail.

#### 2.2.1. SNN-Based Ground-View Event Camera Motion Estimation

The SNN-based ground-view event camera motion estimation module constitutes one of the two core components of the proposed framework. The module adopts the SNN architecture and training procedure developed in our previous study on ground-view event-camera-based motion estimation [23]. For completeness and to clarify the implementation of the motion-estimation branch, its essential architecture and training configuration are summarized below. In the present framework, the pretrained estimator is integrated so that its predictions serve both as the online ground-view camera motion output and as inputs to the subsequent online target-camera orientation calibration and motion-refinement process. The objective of this module is to estimate the motion of the ground-view event camera from event data captured from the ground surface. As shown in the system framework, the event stream of the ground-view event camera is temporally aligned with the target-camera images by using the image acquisition events of the target camera as synchronization markers. Based on these markers, the event stream between two consecutive target-camera frames is divided into a sequence of contiguous and non-overlapping time windows. In the present study, each time window has a duration of 2 ms, and the events within each window are used as the input to the SNN. By processing these short-window event data sequentially, the module produces online motion estimates of the ground-view camera over time.

As shown in Figure 1, to formulate the motion to be estimated, two coordinate systems are considered: $C_{i}$, attached to the ground-view event camera, and $C_{g}$, defined as the projection of $C_{i}$ onto the ground plane. Let $\Delta T_{g}^{j,j+1}$ denote the motion of $C_{g}$ from time *j* to time j+1. In the coordinate convention adopted in this paper, the motion of the ground-view camera is represented by the translation components parallel to the ground plane along the *x*- and *z*-axes, together with the rotation around the *y*-axis, which is perpendicular to the ground. Accordingly, $\Delta T_{g}^{j,j+1}$ is expressed as

(1) $$\begin{array}{c} \Delta T_{g}^{j,j+1}=\left(x_{g}^{j,j+1},0,z_{g}^{j,j+1},0,\psi_{g}^{j,j+1},0\right), \end{array}$$

where $\left(x_{g}^{j,j+1},z_{g}^{j,j+1},\psi_{g}^{j,j+1}\right)$ represent the ground-parallel motion of $C_{g}$ within a short time interval from time *j* to time j+1, and $\psi_{g}^{j,j+1}$ denotes the rotation angle around the *y*-axis. This motion representation is consistent with the planar motion assumption of a ground vehicle and is directly compatible with the subsequent calibration module. Since the target camera operates at a lower frame rate, the motion used for calibration is defined over the interval from time *k* to time k+1, i.e., $\Delta T_{g}^{k,k+1}$. This motion is calculated using a sequence of incremental motions composed of $\Delta T_{g}^{j,j+1}$ within the corresponding target-camera frame interval.

An event camera asynchronously detects brightness changes at each pixel and generates an event stream composed of events $e_{i}=\left(u_{i},v_{i},t_{i},p_{i}\right)$, where $\left(u_{i},v_{i}\right)$ denotes the pixel location, $t_{i}$ the event timestamp, and $p_{i}\in \left\{+,-\right\}$ the polarity. Before being fed into the network, the raw event stream in each 2 ms time window is converted into an event representation suitable for neural processing. We adopt the event voxel grid representation originally introduced in [25], which preserves the temporal distribution of events within the window. Specifically, for all events ${\left\{e_{i}\right\}}_{i=1}^{N_{e}}$ within one time window, the temporal dimension is discretized into *B* bins over [0,B−1], resulting in an event volume of shape (B,H,W), where *H* and *W* are the event camera resolution. In this work, *B* is set to 10. The resulting event voxel grid is then used as the input to the SNN, and the same process is repeated for every subsequent non-overlapping time window. With the event voxel grid tensor of shape (B,H,W) as input, the SNN directly predicts the incremental motion $\Delta T_{g}^{j,j+1}$, i.e., $\left(x_{g}^{j,j+1},z_{g}^{j,j+1},\psi_{g}^{j,j+1}\right)$, of the projected ground-view camera coordinate system $C_{g}$ for the corresponding time window. In the proposed unified framework, the estimated incremental motions are further composed to obtain $\Delta T_{g}^{k,k+1}$ for the corresponding target-camera frame interval, which is subsequently used in the online target-camera orientation calibration process.

The network employs Recurrent Leaky Integrate-and-Fire (RLIF) neurons [26], in which the recurrent membrane dynamics and recurrent output spikes preserve temporal information across consecutive inputs. It follows a shared-backbone and multi-branch architecture: a convolutional spiking block and max-pooling layer are followed by one Spike-Element-Wise (SEW) residual block and two Downsample SEW (DSEW) residual blocks [27], after which three separate output branches predict $x_{g}$, $z_{g}$, and $\psi_{g}$. The layer-by-layer architecture is summarized in Table 1.

The SNN was trained exclusively on synthetic sequences generated using ESIM [28]. Each sequence was 800 ms long. The training set comprised 5835 sequences from six asphalt scenarios, the validation set comprised 587 sequences from three different asphalt scenarios, and the held-out test set comprised 400 sequences from four additional scenarios. The three subsets were separated by scenario. The training and validation sequences were generated using randomized trajectories containing diverse linear and angular motions. The pretrained estimator was subsequently evaluated on the held-out synthetic test set, which covered linear velocities from 1.0 to 12.0 m/s and angular velocities from 4.5 to 300.0°/s, thereby providing an independent evaluation over a broad range of motion magnitudes. None of the real-vehicle sequences evaluated in this study were used for training or fine-tuning, and there is therefore no overlap between the training and experimental data. The network was trained using the geometric loss developed in our previous study. Rather than directly combining translational and rotational errors using an empirically selected weighting factor, this loss measures the residuals between event coordinates warped using the predicted and ground-truth motions, so that the effects of both motion components are expressed in the same image-coordinate space. Training was performed for 105 epochs using PyTorch 2.2.1 and snnTorch 0.8.1 [26], with the Adam optimizer, a batch size of 16, a learning rate of $5\times 10^{-5}$, and gradient clipping with a global norm of 100. Network states were propagated across consecutive 2 ms event windows, and truncated backpropagation through time was performed after every 10 forward passes, corresponding to 20 ms. Further derivations of the loss and comprehensive evaluations of the estimator are provided in the cited study.

Unlike the geometry-based ground-view motion estimation method adopted in our previous work [21], which recovers metric scale at inference from a measured camera-to-ground height, the SNN learns to predict metrically scaled motion through supervised training. The geometric loss is constructed using metrically scaled ground-truth motion and the corresponding camera-to-ground geometry, so scale information is incorporated during training rather than supplied through a fixed camera height at inference. Accordingly, the network receives only the event voxel grid at inference and directly predicts metrically scaled ground-view motion without using a measured camera height, pitch, or roll. To improve robustness to camera-to-ground pose changes, the synthetic training and validation sequences were generated with camera heights between 0.40 and 0.45 m, and each sequence additionally included height variations of up to 0.04 m. Including these variations during training allows the network to learn a motion-estimation mapping that is not tied to a single fixed camera height, thereby improving its ability to accommodate height changes within the represented range. This capability does not imply invariance to arbitrary height changes outside this range or to aggressive maneuvers that violate the steady-motion assumption of the calibration method. Within its supported range, it improves the robustness of ground-view motion estimation and thereby supports more reliable online target-camera orientation calibration.

In the overall framework, the output of the present module corresponds to the online ground-view camera motion estimate. Since it is directly inferred from the ground-view event stream over short, consecutive time windows, it provides short-term motion estimation results that are continuously updated over time. Here, online indicates that the estimate for the current event window is produced directly by the motion-estimation branch and is available before the subsequent calibration-based refinement; it does not denote a measured wall-clock processing latency. Likewise, the 2 ms window specifies the duration over which events are accumulated and the temporal resolution of the motion estimate, rather than the SNN inference time. As such, the online estimate serves as the immediate motion output of the ground-view sensing branch and constitutes one of the principal outputs of the proposed system. By contrast, the refined ground-view camera motion is obtained only after the subsequent calibration process, where these online motion estimates are further optimized under additional constraints.

#### 2.2.2. Online Target-Camera Orientation Calibration

The online target-camera orientation calibration module is built upon our previous work on online camera orientation calibration [21]. Following the basic formulation of the original method, the calibration process is organized into three steps. First, the target-camera motion and the ground-view camera motion used for calibration are obtained. Second, the orientation of the target camera relative to the ground-view camera projection on the ground is estimated based on the extrinsic-parameter invariance constraint. Third, the orientation of the ground-view camera projection relative to the vehicle is estimated using the nonholonomic constraint. By combining the results of the second and third steps, the orientation of the target camera relative to the vehicle can then be recovered. In the present work, while the overall three-step structure is retained, the second step is further improved through a two-sub-step optimization strategy, which not only improves the calibration accuracy but also yields a more accurate ground-view camera motion estimate, thereby benefiting the subsequent estimation based on the nonholonomic constraint.

**Step 1: Motion estimates used for calibration**

In the first step, the target-camera motion and the ground-view camera motion used for calibration are obtained. The motion of the target camera between two consecutive image frames, denoted as $\Delta T_{a}^{k,k+1}$, is estimated by applying a visual odometry algorithm to the image sequence captured by the target camera. In this work, ORB-SLAM2 [24] is used to obtain the target-camera motion estimate.

The motion of the ground-view camera used for calibration is denoted as $\Delta T_{g}^{k,k+1}$, which is defined over the same time interval as $\Delta T_{a}^{k,k+1}$, i.e., between two consecutive target-camera frames. As described in Section 2.2.1, the SNN-based ground-view event camera motion estimation module directly predicts a sequence of incremental motions $\Delta T_{g}^{j,j+1}$ from short, contiguous, and non-overlapping event time windows of 2 ms. Then, $\Delta T_{g}^{k,k+1}$ is calculated by composing the sequence of incremental motions $\Delta T_{g}^{j,j+1}$ within the corresponding target-camera frame interval. In this way, the ground-view camera motion and the target-camera motion are temporally aligned and can be jointly used in the subsequent calibration process.

**Step 2: Estimation of target-camera orientation relative to the ground**

In the second step, the orientation of the target camera relative to the ground-view camera projection on the ground is estimated under the extrinsic-parameter invariance constraint. When the vehicle moves on a regular flat road in a relatively steady manner, without aggressive maneuvers such as strong acceleration or high-speed turning, the extrinsic parameters of the onboard cameras can be regarded as approximately unchanged during motion. Let $T_{ga}$ denote the pose of the target camera with respect to the ground-view camera projection on the ground. Under this assumption, the motions of the two sensing branches satisfy

(2) $$T_{ga}\Delta T_{a}^{k,k+1}=\Delta T_{g}^{k,k+1}T_{ga},$$

which provides the geometric constraint used in this step.

Because the target camera is monocular and its motion estimate does not contain absolute scale, $T_{ga}$ is represented as a similarity transformation:

(3) $$T_{ga}=\left[\begin{array}{cc} sR_{ga} & t_{ga}\\ 0_{1\times 3} & 1 \end{array}\right],$$

where *s* is the scale factor, $R_{ga}$ is the rotation matrix, and $t_{ga}$ is the translation vector. For convenience, the scaled rotation term $sR_{ga}$ is denoted by $R_{ga}^{s}$.

In the original formulation of this calibration method [21], the estimation of $R_{ga}^{s}$ and the refinement of $\Delta T_{g}^{k,k+1}$ were performed in a coupled manner. In the present work, this step is reformulated as a two-sub-step procedure. The rationale is that errors in the translational component of $T_{ga}$ can adversely affect the coupled optimization. To mitigate this influence, the rotational part is first optimized separately, so that the ground-view camera motion can be refined under a cleaner geometric relationship. The scale-related estimation is then carried out on top of the improved rotational alignment.

*Sub-step 2-1: Rotation-based optimization*

The first sub-step focuses on the rotational components of the two motion estimates. Since the target-camera motion is usually more reliable due to the broader field of view of the target camera, its rotational information is used to improve both $R_{ga}$ and the rotational part of the ground-view camera motion. The corresponding rotation constraint is written as

(4) $$R_{ga}\Delta R_{a}^{k,k+1}=\Delta R_{g}^{k,k+1}R_{ga}.$$

Based on this relationship, the residual for the *k*-th motion pair is defined by

(5) $$e_{k,k+1}^{R}=\log\left({\left(\Delta R_{a}^{k,k+1}\right)}^{-1}R_{ga}^{-1}\Delta R_{g}^{k,k+1}R_{ga}\right),$$

and the corresponding objective function is

(6) $$f_{R}=\sum_{k=0}^{n-1}{\left(e_{k,k+1}^{R}\right)}^{⊤}\Omega_{k,k+1}e_{k,k+1}^{R},$$

where $\Omega_{k,k+1}$ is the information matrix associated with the measurement $\Delta R_{a}^{k,k+1}$. Here, k=0,1,…,n−1 because the calibration sequence contains n+1 target-camera frames, from which *n* relative motions are obtained. By minimizing this objective, $R_{ga}$ and $\Delta R_{g}^{k,k+1}$ are jointly updated. The optimization is solved using g2o [29].

*Sub-step 2-2: Estimation of $R_{ga}^{s}$*

After the rotational quantities have been refined, the second sub-step estimates $R_{ga}^{s}$ within the similarity-transformation formulation. Here, the refined $R_{ga}$ and $\Delta R_{g}^{k,k+1}$ obtained in Sub-step 2-1 are used to construct the improved rotational alignment, while $\Delta T_{g}^{k,k+1}$ is treated as fixed in the present sub-step.

Based on the same geometric constraint in eq:step2handeye, the residual is defined as

(7) $$e_{k,k+1}^{T}=\log\left({\left(\Delta T_{a}^{k,k+1}\right)}^{-1}T_{ga}^{-1}\Delta T_{g}^{k,k+1}T_{ga}\right),$$

and the corresponding objective function is

(8) $$f_{T}=\sum_{k=0}^{n-1}{\left(e_{k,k+1}^{T}\right)}^{⊤}\Omega_{k,k+1}e_{k,k+1}^{T},$$

where $\Omega_{k,k+1}$ denotes the information matrix associated with the target-camera motion measurement $\Delta T_{a}^{k,k+1}$. As in Sub-step 2-1, the summation index runs from 0 to n−1 because *n* relative motions are obtained from a calibration sequence containing n+1 target-camera frames. By minimizing this objective, the scaled rotation term $R_{ga}^{s}$ is estimated on the basis of the refined rotational alignment obtained in Sub-step 2-1.

Overall, the proposed two-sub-step strategy improves the calibration process by reducing the adverse influence of translation errors in $T_{ga}$ on the refinement of the ground-view camera motion. By first refining the rotational relationship and then performing the scale-related estimation, the proposed method leads to more accurate target-camera orientation calibration while simultaneously improving the ground-view camera motion estimate. In the overall framework, the refined ground-view camera motion should be distinguished from the online ground-view camera motion estimate produced directly by the SNN-based motion estimation module. The online motion estimate provides immediately available and continuously updated motion information over short time intervals, making it suitable for representing the instantaneous motion of the ground-view camera. By contrast, the refined ground-view camera motion is obtained after further incorporating the target-camera motion and the geometric constraints in the calibration process, and is intended to provide a more accurate motion estimate for use at the system level. Moreover, because the refined motion is still derived from the ground-view branch, it preserves absolute scale while achieving improved accuracy through optimization. Therefore, it offers a more favorable form of motion estimate than typical monocular motion estimation methods, whose outputs generally do not directly provide absolute scale.

**Step 3: Estimation of ground-view camera yaw angle relative to the vehicle**

In the third step, the relative orientation between $C_{g}$ and $C_{v}$, denoted by $R_{vg}$, is estimated using the nonholonomic constraint of the vehicle. The incremental motion of the vehicle, $\Delta T_{v}^{k,k+1}$, is related to the ground-view camera motion by

(9) $$\Delta T_{v}^{k,k+1}=T_{vg}\Delta T_{g}^{k,k+1}T_{vg}^{-1}.$$

Since the vehicle motion is constrained to the ground plane, both $\Delta T_{v}^{k,k+1}$ and $\Delta T_{g}^{k,k+1}$ can be treated as planar motions. Without loss of generality, $C_{v}$ and $C_{g}$ are rotated such that their *x*-axes point to the front and *y*-axes point to the left compared to their original orientation. Then, $R_{vg}$ can be regarded as a rotation with angle $\theta_{vg}$ around the axis perpendicular to the ground plane. Let

$$\Delta T_{g}^{k,k+1}=\left(x_{g}^{k,k+1},y_{g}^{k,k+1},\theta_{g}^{k,k+1}\right),\;T_{vg}=\left(x_{vg},y_{vg},\theta_{vg}\right),$$

then the nonholonomic constraint can be written as

(10) $${\dot{x}}_{v}^{k,k+1}\sin\theta_{v}^{k,k+1}-{\dot{y}}_{v}^{k,k+1}\cos\theta_{v}^{k,k+1}=0,$$

where $\Delta T_{v}^{k,k+1}=\left(x_{v}^{k,k+1},y_{v}^{k,k+1},\theta_{v}^{k,k+1}\right)$.

Because the camera is rigidly mounted on the vehicle, the rotational increments represented in $C_{g}$ and $C_{v}$ satisfy

(11) $$\theta_{v}^{k,k+1}=\theta_{g}^{k,k+1}.$$

Taking into account the errors in $\Delta T_{g}^{k,k+1}$ and $T_{vg}$, the residual associated with the nonholonomic constraint is expressed as

(12) $$\begin{array}{cc} e_{k,k+1}\left(\theta_{vg}\right) & =\;{\dot{x}}_{v}^{k,k+1}\sin\theta_{v}^{k,k+1}-{\dot{y}}_{v}^{k,k+1}\cos\theta_{v}^{k,k+1}\\ \; & =\;{\dot{y}}_{g}^{k,k+1}\cos\left(\theta_{vg}-\theta_{g}^{k,k+1}\right)+{\dot{x}}_{g}^{k,k+1}\sin\left(\theta_{vg}-\theta_{g}^{k,k+1}\right)+{\dot{\theta }}_{g}^{k,k+1}x_{vg}. \end{array}$$

Assuming that ${\dot{x}}_{g}^{k,k+1}$, ${\dot{y}}_{g}^{k,k+1}$, and ${\dot{\theta }}_{g}^{k,k+1}$ remain constant within each short interval, $\theta_{vg}$ is estimated by minimizing

(13) $$\theta_{vg}=\underset{\theta_{vg}}{\arg\min}\sum_{k=0}^{n-1}e_{k,k+1}^{2}.$$

This least-squares problem is solved using g2o [29]. Because the ground-view camera motion has already been refined in Step 2, the motion input used here is more accurate, which further improves the estimation of $\theta_{vg}$. Finally, by combining the results obtained in Steps 2 and 3, the orientation of the target camera relative to the vehicle can be recovered.

## 3. Experiments

### 3.1. Experimental Setup

To evaluate the proposed framework in real-world driving scenarios, three cameras were installed on the vehicle, as shown in Figure 3: a ground-view event camera, a ground-view high-speed frame-based camera, and a front-view frame-based target camera. The ground-view event camera was a DAVIS346 (iniVation Inc., Zurich, Switzerland) with a resolution of 346 × 260. The ground-view high-speed frame-based camera was an MQ013MG-ON (XIMEA Inc., Münster, Germany) operating at 500 Hz with a resolution of 640 × 512. The front-view target camera was also an MQ013MG-ON (XIMEA Inc.), operating at 50 Hz with a resolution of 1280 × 1024. Among the three cameras, the ground-view high-speed frame-based camera was additionally used to generate reference motion for quantitative evaluation of the ground-view motion estimates. Specifically, the synchronized image sequence captured by this camera was processed by HSV-odometry [30], which was adopted in our previous calibration study [21]. The resulting motion estimates were then used as reference for comparison with the proposed method. HSV-odometry jointly estimates planar translation and yaw rotation. The underlying method was previously evaluated against an OptiTrack motion-capture system in controlled experiments, where submillimeter mean errors were reported for short-interval travel-distance estimation [30]. This independent validation, together with the semi-dense formulation and the high frame rate used in the present setup, supports its use for generating short-interval reference motion. However, HSV-odometry itself is still not well suited for real-world deployment. First, the high frame rate leads to a substantial increase in data volume and computational cost. Second, for HSV-odometry, recovering motion with absolute scale requires prior knowledge of the camera pose relative to the ground. Therefore, although HSV-odometry was used in this study to provide reference motion, these limitations still prevent it from being a practical solution for real-world applications.

Offline calibration determined the pose of the ground-view event camera relative to the ground, with the camera positioned 0.43 m above the ground at a pitch angle of $-1.3^{\circ }$ and a roll angle of $-1.9^{\circ }$. The relative extrinsic parameters among the three cameras, as well as the ground-truth extrinsic parameters of the front-view target camera with respect to the vehicle, were also obtained offline through a dedicated calibration procedure. Specifically, the camera-to-camera extrinsics were first calibrated in the laboratory with the aid of an OptiTrack motion-capture system. The extrinsic parameters of the front-view target camera relative to the vehicle were then determined through an additional geometry-based calibration process. To align the measurements from the three sensors in time, the target camera was used as the timing reference. Each exposure of the target camera generated a synchronization signal shared with the two ground-view cameras. In the event stream, this signal was recorded as a dedicated marker, which allowed the events lying between two successive target-camera frames to be partitioned into disjoint short temporal segments for motion estimation. Meanwhile, the same trigger was used for the high-speed frame-based camera so that its images were temporally aligned with the other sensing modalities.

The real-vehicle evaluation used 12 sequences collected by driving the vehicle on asphalt roads along both straight and curved trajectories within a university campus. Each sequence had a duration of 10 s, yielding 120 s of data in total. The linear speed of the ground-view event camera ranged from approximately 1.0 to 5.7 m/s, corresponding to approximately 3.6 to 20.5 km/h, while the magnitude of its angular velocity was predominantly between about 5 and $50^{\circ }/s$. The campus-road environment limited the attainable vehicle speed, and the reported linear-speed range therefore reflects the constraints of the data-collection site. It was not selected as a representative speed range for practical road vehicles and should not be interpreted as the upper operating-speed limit of the framework. The present experiments therefore evaluate the complete framework under the low-speed driving conditions covered by these campus-road sequences. A representative example of the ground-view event-camera data is presented in Figure 4, where both an event image and a grayscale frame are shown.

### 3.2. Evaluation of Ground-View Motion Estimation

Based on the offline calibration described in the experimental setup, the relative pose between the two ground-view cameras and their poses with respect to the ground were obtained beforehand. Using these calibrated poses, the motion estimated from the ground-view high-speed frame-based camera was transformed into the coordinate system of the ground-view event camera to provide reference motion. The proposed method was compared with PEME [31], an event-based motion estimation method that progressively aligns sampled events in a batch to a time-surface map and updates the motion model by minimizing a time-surface loss. The comparison was conducted on all 12 sequences. Since the motion estimation methods predict short-interval motion, the corresponding velocities $\left(v_{xg},v_{zg},\omega_{\psi g}\right)$ were computed under the constant-velocity assumption, which is justified by the short duration of each time window, and the RMSEs of these velocity components were used as the evaluation metrics. Since PEME can estimate only the motion of $C_{i}$ and does not directly provide absolute scale, the calibrated pose of the event camera relative to the ground was used to convert its estimation results into metrically scaled $\left(v_{xg},v_{zg},\omega_{\psi g}\right)$. The calibrated camera-to-ground poses used to construct the reference motion and metrically scale the PEME results were not supplied to the SNN, whose inference input consisted only of event voxel grids. For the SNN-based ground-view event camera motion estimation module, the 2 ms motion estimates were further aggregated over every ten consecutive time windows to produce 20 ms motion estimates, thereby matching the time interval of each refined ground-view camera motion estimate. For each method and motion component, the RMSE was calculated separately for each sequence. The values reported in Table 2 are the arithmetic mean and sample standard deviation of the resulting 12 sequence-wise RMSEs.

The quantitative results are summarized in Table 2, and a representative example is shown in Figure 5. For the translational velocity components $v_{xg}$ and $v_{zg}$, both the proposed online estimate and the proposed refined estimate yield lower mean sequence-wise RMSEs than PEME. Specifically, for $v_{xg}$, the mean sequence-wise RMSE is reduced from 0.3364 m/s for PEME to 0.2175 m/s for the proposed online estimate and 0.2195 m/s for the proposed refined estimate. For $v_{zg}$, the mean sequence-wise RMSE decreases from 0.3845 m/s for PEME to 0.3197 m/s and 0.3215 m/s, respectively. These results indicate that the proposed SNN-based motion estimation module can provide more accurate translational motion estimates from the ground-view event stream. In these two components, the refined estimate remains at a similar accuracy level to the online estimate, with only slight increases in the mean sequence-wise RMSE.

For the angular velocity component $\omega_{\psi g}$, the proposed online estimate exhibits a relatively large mean sequence-wise RMSE of 44.9615°/s, whereas the proposed refined estimate reduces this to 7.4374°/s, which is not only substantially lower than that of the online estimate but also lower than the 13.1399°/s obtained by PEME. The relatively large angular error of the online estimate is consistent with the real-world outdoor results reported in our previous comprehensive evaluation of the SNN-based motion estimator [23]. In that study, the same estimator exhibited substantially lower angular errors in the synthetic and indoor evaluations, whereas a larger angular error occurred in the outdoor evaluation. This cross-condition difference suggests that one possible reason is the synthetic-to-real gap: the SNN was trained exclusively on synthetic data, while real-world outdoor data contain disturbances that are not fully represented in the simulation, such as shadows caused by the vehicle body and other appearance and sensing variations. These factors may affect the event patterns and consequently degrade the estimation accuracy. Fine-tuning the pretrained SNN on representative real-world outdoor event data may help reduce this domain gap and improve angular velocity estimation accuracy. Nevertheless, this result for the angular component should not be considered in isolation from the overall capability of the SNN-based motion estimator. As shown by the translational results, the online estimate achieves lower mean sequence-wise RMSEs than PEME for both translational components. It also directly provides metrically scaled motion estimates from short event windows and is designed to tolerate small camera-to-ground height variations.

The improvement in the rotational component results from the complementary roles of the target-camera motion and the proposed optimization strategy. The target-camera motion estimated by ORB-SLAM2 provides more reliable rotational information, while the rotation constraint and the proposed two-sub-step optimization strategy use this information to jointly update $R_{ga}$ and $\Delta R_{g}^{k,k+1}$. Thus, the target-camera motion serves as the information source, and the optimization strategy provides the mechanism for incorporating this information into the refinement, rather than the two acting as separable alternatives. By retaining the advantages of the online SNN-based motion estimate while further improving its rotational component, the proposed framework enables the motion estimator to contribute more effectively to the overall motion-estimation and calibration system. The same tendency can also be observed in Figure 5. The proposed online estimate follows the reference motion more closely than PEME in the translational components, while the refined estimate provides a clear improvement for the angular component and remains close to the reference in translation. No additional temporal filtering or sample-level outlier rejection was applied to the synchronized motion estimates before plotting or RMSE calculation, and all samples were retained in the evaluation. Consequently, the curves retain both short-term vehicle-motion variations and method-specific estimation fluctuations. Since the velocities were calculated from relative motions over 20 ms intervals, localized pose-estimation variations are more visually pronounced in the velocity curves. Peaks appearing simultaneously in multiple curves may correspond to transient vehicle motion, whereas peaks confined to an individual curve may reflect method-specific estimation fluctuations. These results demonstrate that the proposed framework not only enables online ground-view motion estimation from the event stream but also further improves the motion estimate through the subsequent calibration process. In particular, after refinement, the framework provides a more accurate ground-view motion estimate with absolute scale, which is advantageous compared with typical monocular motion estimation methods whose outputs do not directly contain scale.

### 3.3. Evaluation of Online Target-Camera Orientation Calibration

To obtain reliable target-camera motion for calibration, ORB-SLAM2 [24] was used to estimate the trajectory of the front-view camera. The calibration performance was evaluated on the collected 12 sequences by comparing the absolute errors of the rotation angles extracted from the target-camera extrinsic parameters estimated by the baseline method SensorX2car [4], the original method [21], and the proposed method, with respect to the corresponding ground-truth rotation angles obtained in Section 3.1. SensorX2car is a state-of-the-art online orientation calibration approach based on vanishing-point detection. To avoid the influence of ORB-SLAM2 initialization, the first 0.7 s of each sequence were discarded, so that 9.3 s of data from each 10 s sequence were used in the evaluation. Since SensorX2car produces one calibration result from each input image independently, its final output for a given sequence was obtained by averaging the image-wise calibration results over the whole sequence.

For both the original method and the proposed method, the same experimental configuration was adopted. Each valid sequence was divided into multiple overlapping sub-sequences of 9 s. The motion estimates from the front-view target camera, operating at 50 Hz, were synchronized with those from the ground-view event camera. In the event-based branch, motion was first estimated over 2 ms event windows, yielding a temporal resolution of 0.002 s, and was then converted to the same 0.02 s interval as the target-camera motion estimates for temporal alignment. Each sub-sequence therefore consisted of synchronized motion estimates with a time step of 0.02 s. For evaluation, successive 9 s sub-sequences were offset by one 0.02 s time step so that all available intervals were included. Calibration was performed independently on each sub-sequence, and the absolute yaw, pitch, and roll errors obtained from the resulting calibration estimates were averaged to form the sequence-level errors. This overlapping-window construction defines the evaluation samples and does not imply continuous or frame-by-frame execution of the calibration procedure. Since both methods require initial estimates of the camera extrinsics, a common set of noisy target- and ground-view-camera extrinsics was generated by independently perturbing their ground-truth transformations with random rotation and translation errors. The perturbation magnitudes were set to 5° for rotation and 0.03 m for translation. To generate the rotation perturbation, a random three-dimensional vector was first sampled and normalized to define a rotation axis, and its sign was randomly assigned so that the resulting rotation angle became either $+5^{\circ }$ or $-5^{\circ }$. For the translation perturbation, a translation vector was constructed such that each of its components had an absolute value of 0.03 m, while the sign of each component was assigned randomly. These perturbations were then applied to the ground-truth extrinsic matrices to obtain perturbed initial transformations. The resulting initial values model discrepancies between the available extrinsic estimates and the current camera extrinsics, such as those that may arise when previously calibrated camera parameters become inaccurate after a change in camera installation. In total, ten pairs of noisy target- and ground-view-camera extrinsic estimates were generated and shared across all sequences. For each sequence, both the original method and the proposed method were executed ten times using these different initial values. For these two methods, the sequence-level values reported in Table 3 are the arithmetic means of the corresponding trial-level errors over the ten trials. For all three methods, the final Mean row is the arithmetic mean of the 12 sequence-level values.

Table 3 summarizes the absolute errors of the yaw, pitch, and roll angles of the target-camera orientation estimated by SensorX2car, the original method, and the proposed method on the twelve real-vehicle sequences. For the yaw angle, whose calibration accuracy is especially important for maintaining accurate alignment between the target camera and the vehicle coordinate system in ground-vehicle applications, the proposed method shows a clear advantage. SensorX2car achieves the smallest yaw error on Sequence 2, whereas the proposed method achieves the smallest yaw error on the other eleven sequences and yields smaller yaw errors than the original method on all twelve sequences. Moreover, the overall mean yaw error of the proposed method is 0.2551°, which is substantially lower than those of SensorX2car and the original method, namely 2.3440° and 2.4331°, respectively. These results show that the proposed improvement provides a pronounced reduction in yaw calibration error.

For the pitch angle, the proposed method yields a lower error than the original method on eleven of the twelve sequences and reduces the overall mean error from 0.2844° to 0.2358°. Although the proposed method achieves the smallest pitch error on four sequences, SensorX2car achieves the smallest error on the other eight sequences and the lowest overall mean error of 0.1978°. For the roll angle, the proposed method remains close to the original method, with overall mean errors of 1.3132° and 1.3576°, respectively, and yields a lower error on seven of the twelve sequences. SensorX2car nevertheless achieves the smallest roll error on ten sequences and the lowest overall mean error of 0.6908°. Overall, the proposed method provides the most favorable trade-off among the compared methods for target-camera orientation calibration on real-vehicle data, chiefly because it achieves markedly higher yaw accuracy while maintaining pitch and roll accuracy close to those of the original method. Although SensorX2car attains lower overall mean errors in pitch and roll, the pronounced yaw advantage of the proposed method is especially important for ground-vehicle applications.

To examine whether the mean errors reported in Table 3 conceal sensitivity to the initial extrinsic estimates, Table 4 reports the sample standard deviation and range of the corresponding ten trial-level errors for the original and proposed methods, both of which require initial extrinsic estimates, under the same 5°/0.03 m perturbation setting.

The proposed method yields a lower yaw standard deviation than the original method on eleven of the twelve sequences; its sequence-wise yaw standard deviations range from 0.0155° to 0.0598°, compared with 0.0254° to 0.2355° for the original method. This indicates that the improved calibration procedure not only reduces the mean yaw error but also generally reduces its sensitivity to the initial extrinsic estimates. The proposed method exhibits greater trial-to-trial variation in pitch and roll, with maximum standard deviations of 0.0163° and 0.1373°, respectively. Nevertheless, the reported ranges indicate that the greater pitch and roll variability does not arise from a single exceptionally large trial-level error.

To further evaluate the sensitivity of the proposed method to the magnitude of the initial extrinsic errors, four paired rotation/translation perturbation levels were considered: 2°/0.01 m, 5°/0.03 m, 10°/0.05 m, and 15°/0.07 m. The same ten random perturbation-direction templates used in Table 3 and Table 4 were retained across all four levels. For each trial, the rotation axis, the sign of the rotation angle, and the signs of the three translation components were kept unchanged for both the target and ground-view cameras, while only the absolute rotation angle and translation-component magnitude were varied. This paired design separates the effect of perturbation magnitude from variation caused by resampling the perturbation directions. At each perturbation level, the trial-level errors were calculated as described above. The ten trial-level errors were then averaged for each sequence, and Table 5 reports the arithmetic mean of the resulting twelve sequence-level errors.

As the perturbation level increases from 2°/0.01 m to 15°/0.07 m, the mean yaw error increases gradually from 0.2412° to 0.2717°. In contrast, the mean pitch error remains between 0.2354° and 0.2367°, while the mean roll error remains between 1.3132° and 1.3261° without a monotonic increase. These results indicate that the mean orientation calibration accuracy remains largely stable over the evaluated perturbation levels, although the yaw component exhibits greater sensitivity to the magnitude of the initial extrinsic errors than the pitch and roll components.

## 4. Discussion

The experimental results over twelve synchronized real-vehicle sequences demonstrate the effectiveness of the proposed unified framework from two aspects. First, in the evaluation of ground-view motion estimation, the proposed framework produced more accurate translational motion estimates than PEME, and the refined ground-view motion obtained through the calibration process further improved the rotational component compared with the online estimate. These results indicate that the framework is able not only to provide online motion estimates directly from short event windows but also to improve the motion estimate by incorporating the target-camera motion and geometric constraints. Second, in the evaluation of online target-camera orientation calibration, the proposed method achieved a markedly lower yaw error than both SensorX2car and the original method. For pitch, the proposed method improved upon the original method but did not outperform SensorX2car. For roll, the proposed method remained close to the original method, whereas SensorX2car yielded the lowest mean error. The markedly reduced yaw error is particularly important because, for ground vehicles, yaw calibration more directly affects the alignment between the target camera and the vehicle coordinate system, which in turn influences a wide range of downstream tasks. Overall, the results support the advantage of integrating ground-view event-based motion estimation and online calibration in a unified framework, through which the motion estimate is refined and the target-camera orientation is estimated more accurately.

The initialization analyses further clarify the behavior of the calibration method. Under the baseline perturbation setting, the proposed method produced a lower yaw-error standard deviation than the original method on eleven of the twelve sequences, indicating that the yaw improvement was generally less sensitive to the tested initial extrinsic estimates. Although the proposed method exhibited greater trial-to-trial variation in pitch and roll, the corresponding error ranges did not reveal an isolated exceptionally large trial-level error. Across the four evaluated perturbation levels, its mean pitch and roll errors remained nearly unchanged, while the mean yaw error increased only gradually as the perturbation magnitude increased. Taken together, these results demonstrate an additional practical advantage of the proposed method: within the evaluated range, it maintains largely stable mean orientation calibration accuracy as the magnitude of the initial extrinsic errors increases.

At the same time, this study still has several limitations. The proposed framework relies on sufficiently informative ground-surface events, and its performance may degrade in environments where the ground texture is weak or the surface condition is unfavorable. Nevertheless, the experimental results on real asphalt-road data indicate that the framework can still achieve satisfactory accuracy on this common road surface. The method also depends on the quality of the target-camera motion estimated by the visual odometry algorithm, and failures or drift in that estimation will affect the final calibration result. This issue is alleviated to some extent in the proposed framework, since the calibration process makes use of multiple motion estimates rather than relying on a single estimate alone. The real-vehicle evaluation is also limited to low-speed sequences collected on campus roads. Consequently, these experiments do not establish the performance of the complete framework at higher road-vehicle speeds. Although the SNN-based motion-estimation branch was evaluated over a broader motion range on the held-out synthetic test set, this component-level evidence does not replace higher-speed synchronized real-vehicle evaluation of the complete framework. Future work will extend the evaluation of the complete framework to higher vehicle speeds using synchronized real-vehicle data.

Although computational efficiency is important for practical deployment, this study focuses on developing the proposed framework and evaluating its performance in ground-view motion estimation, calibration-based motion refinement, and target-camera orientation calibration. A systematic investigation of the computational efficiency of the complete framework, including implementation optimization and computational-performance evaluation, is outside the research scope of this study and is therefore left for future work. At present, the computational performance of the complete framework cannot yet be assessed in a representative manner because its two core components require different forms of implementation development before they can be evaluated together as an integrated system. For online calibration, the current implementation has not yet been parallelized or optimized for execution speed and is therefore not yet ready for direct deployment. However, camera extrinsic parameters generally remain stable over short periods, so online calibration does not require frame-by-frame updates at the rate demanded by time-critical perception tasks. This lower update-frequency requirement makes the absence of execution-speed optimization less critical at the current stage. Nevertheless, parallelization and execution-speed optimization remain necessary for practical deployment and will be pursued in future work. For the SNN-based motion estimator, the present difficulty in obtaining a representative computational evaluation instead arises from the computing platform. The network is currently implemented using PyTorch and snnTorch and has not been deployed on neuromorphic hardware; consequently, the current software implementation does not fully exploit the sparse, event-driven computation targeted by SNNs. Its measured inference time would depend strongly on the software framework and hardware platform and would not provide a representative measure of the performance attainable through neuromorphic deployment. Accordingly, this study regards neuromorphic deployment and direct evaluation of computational latency and energy efficiency as necessary future work. The potential benefits of such a deployment are supported by previous studies demonstrating low-latency and energy-efficient execution of sparse, event-driven SNN workloads on neuromorphic platforms [32,33], including an event-based automotive classification task implemented on Loihi [34]. In addition, the end-to-end computational performance of the complete framework depends not only on the individual processing times of the SNN estimator, target-camera visual odometry, and calibration optimization but also on the synchronization and data scheduling among these components. Future work will therefore include parallelizing and optimizing the calibration procedure, deploying the SNN estimator on neuromorphic hardware, integrating the complete processing pipeline on a clearly specified platform, and systematically evaluating computational latency, energy efficiency, calibration update frequency, and the time required to obtain the first valid calibration result.

## 5. Conclusions

This paper presented a unified framework for ground-view event-camera-based motion estimation and online target-camera orientation calibration for ground vehicles. In the proposed framework, the motion of the ground-view event camera is first estimated online from the event stream and is then further refined during calibration by incorporating the target-camera motion and geometric constraints. The resulting online estimate provides direct short-term motion information from the ground-view sensing modality, while the refined estimate provides a more accurate motion result after system-level optimization. In addition, the online calibration method was improved so that the ground-view motion can be further optimized together with the calibration variables, leading to both a refined motion estimate and more accurate target-camera orientation calibration. Experimental results on real-vehicle sequences demonstrated that the proposed framework provides more accurate translational ground-view motion estimates and further improves the rotational component through motion refinement. The calibration results showed a marked advantage in yaw, whose accuracy is especially important for maintaining alignment between the target camera and the vehicle coordinate system in ground-vehicle applications. For pitch and roll, the proposed method maintained mean accuracy close to the original method, while SensorX2car achieved lower mean errors in these components. Beyond these mean-error comparisons, under the baseline initialization setting, the proposed method yielded lower yaw-error variability than the original method on eleven of the twelve sequences. Across the four evaluated initial perturbation levels, its mean orientation errors remained largely stable, demonstrating robustness to the magnitude of the initial extrinsic errors within the evaluated range. These results confirm the effectiveness of the proposed unified framework in combining ground-view event-camera-based motion estimation and online target camera orientation calibration. The real-vehicle validation in this study was conducted on asphalt roads. Future work will extend the evaluation to broader road and illumination conditions and a wider range of vehicle motions. Longer continuous driving sequences will also be used to assess long-term calibration stability.

## Figures and Tables

**Figure 1:**
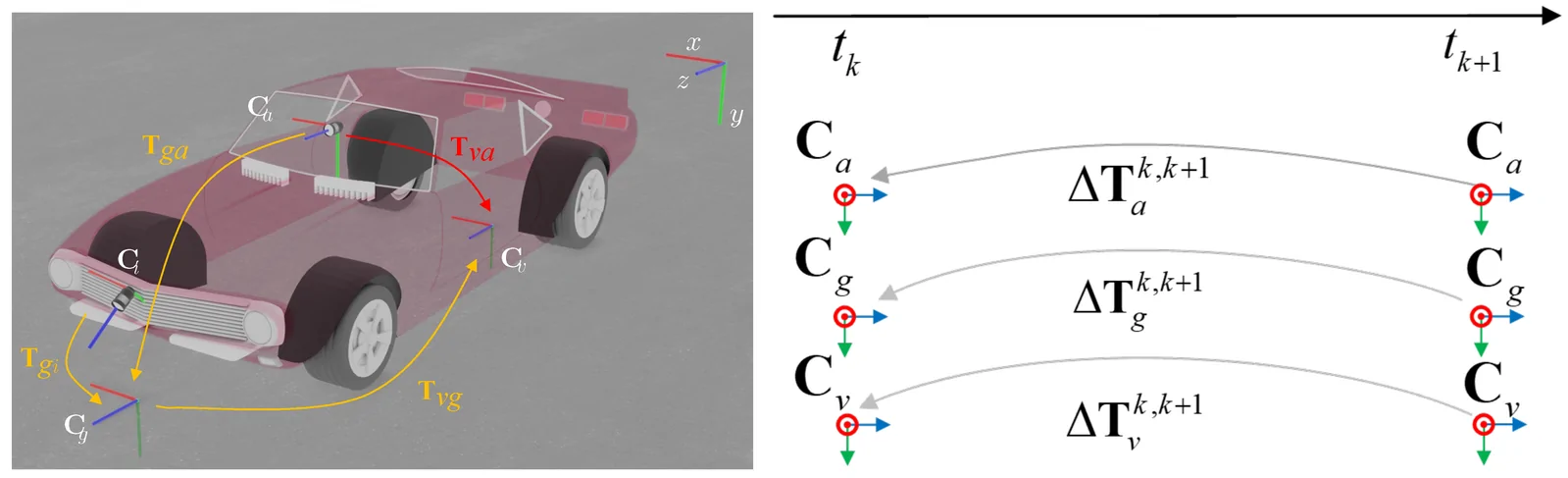
Geometry of the problem with temporal transformations from time *k* to time k+1 and extrinsic transformations.

**Figure 2:**
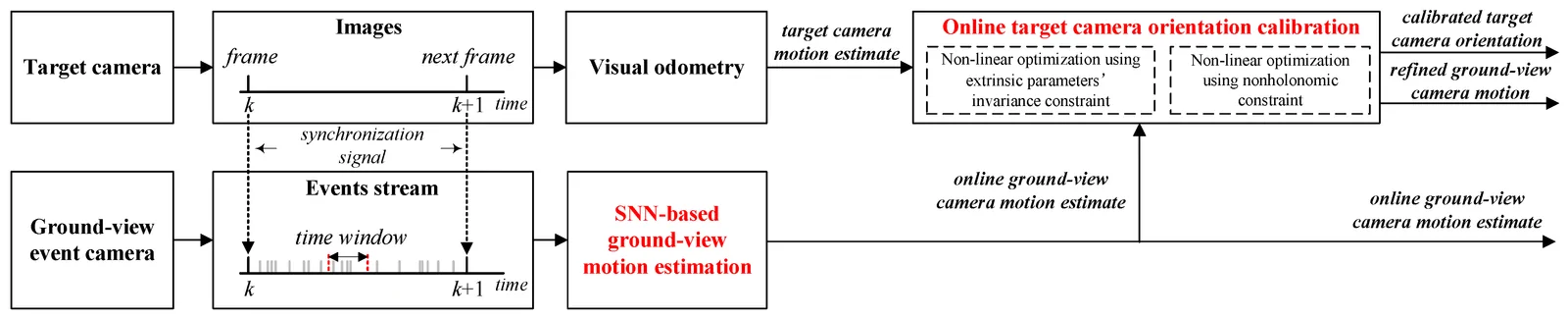
Overall framework of the proposed unified system. The two core modules emphasized in this study, namely SNN-based ground-view motion estimation and online target-camera orientation calibration, are highlighted in red.

**Figure 3:**
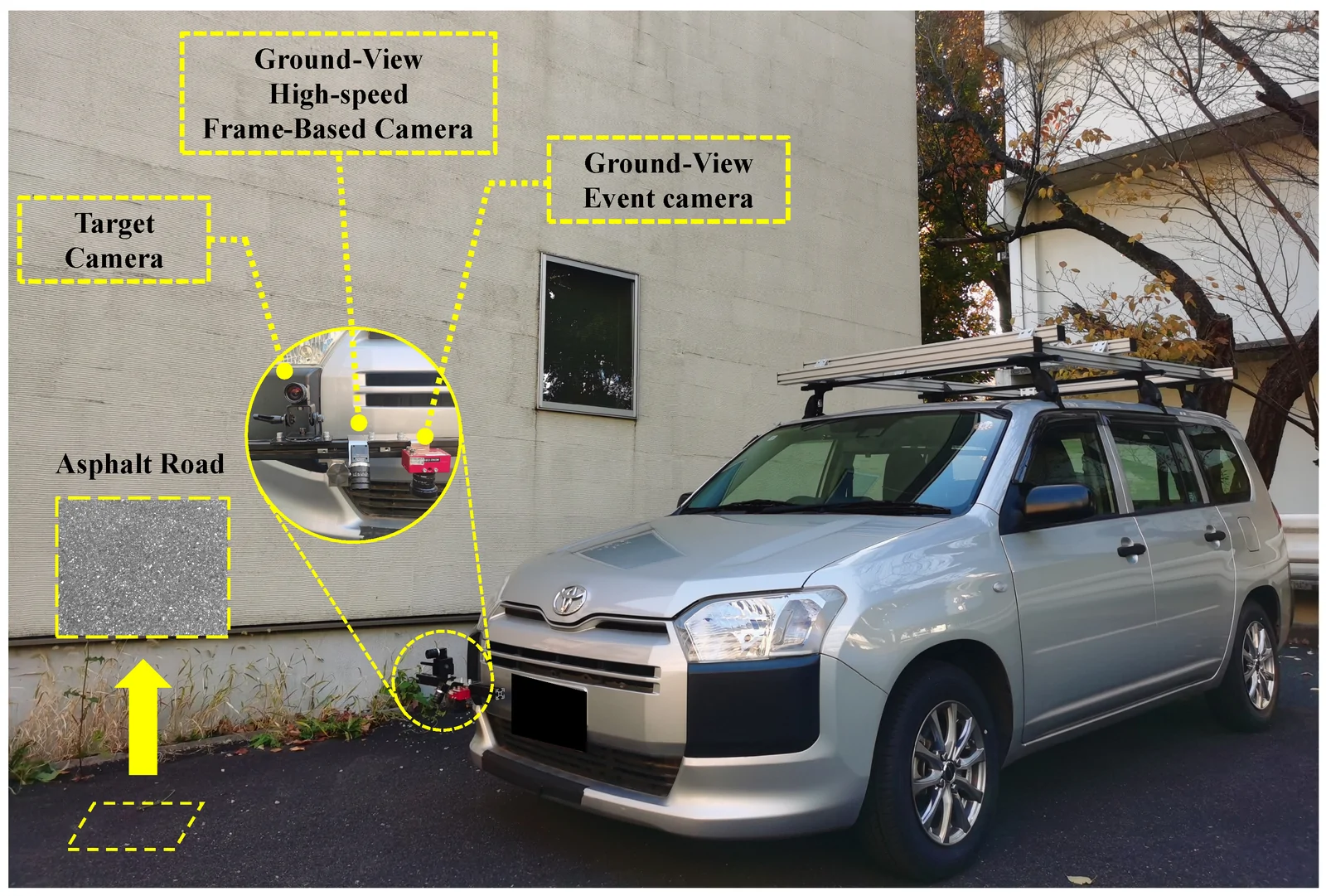
The experimental vehicle equipped with a ground-view event camera, a ground-view high-speed frame-based camera, and a front-view target camera, together with the asphalt road texture used for data collection in the experiments.

**Figure 4:**
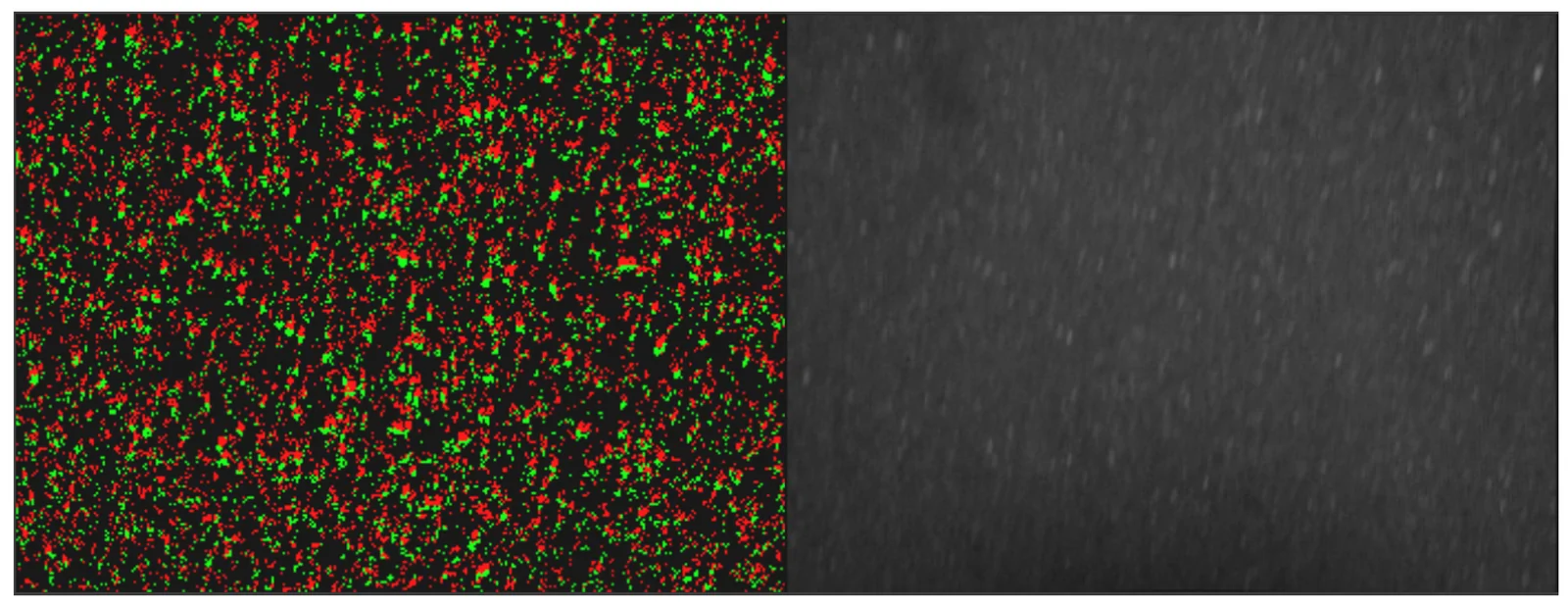
Sample data collected by the DAVIS346 ground-view event camera on an asphalt road. The event frame is on the left and the grayscale image is on the right. The brightness of the displayed images has been enhanced only for visualization.

**Figure 5:**
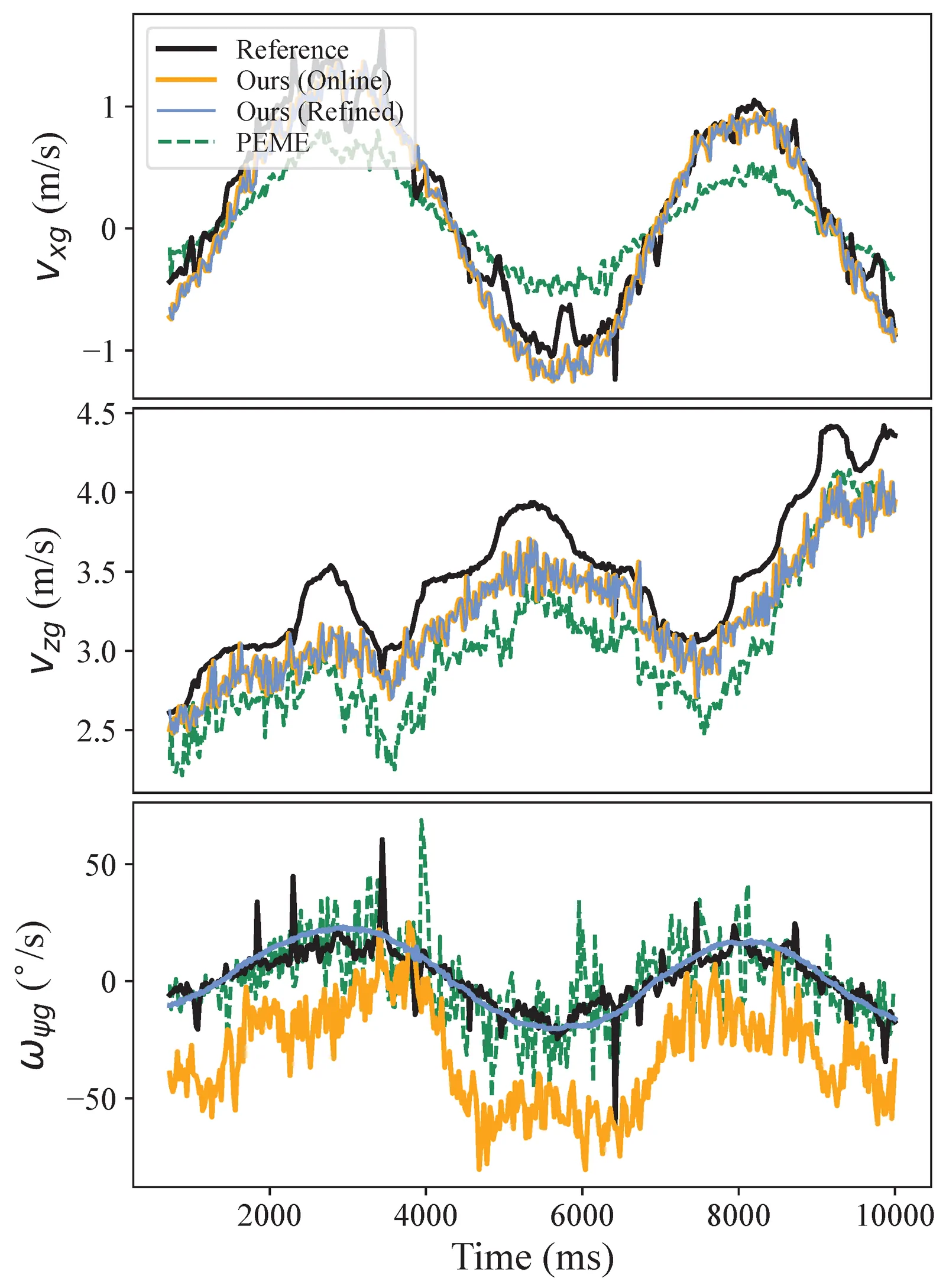
Comparison of ground-view motion estimation results on a real-vehicle data sequence.

**Table 1:** Layer-by-layer architecture of the SNN-based ground-view motion estimator.

Stage or Element	Configuration	Result or Role
Event representation	Events in each 2 ms window are encoded as a voxel grid with B=10 temporal bins.	Input tensor of shape (B,H,W)=(10,260,346).
Spiking neuron	RLIF with trainable membrane decay, a recurrent 3×3 convolution, subtractive reset, and an arctangent surrogate gradient.	Used throughout the convolutional spiking blocks to retain temporal information.
Initial feature extraction	7×7 convolution (10→64 channels, stride 2), batch normalization, RLIF, and 3×3 max pooling (stride 2).	Produces a 64-channel feature map.
Shared residual stage 1	One SEW block with two convolutional spiking blocks and a shortcut connection; the channel number remains 64.	Extracts shared features without spatial downsampling.
Shared residual stage 2	One DSEW block with stride-2 downsampling and a channel transition from 64 to 128.	Produces a downsampled 128-channel feature map.
Shared residual stage 3	One DSEW block with stride-2 downsampling and a channel transition from 128 to 256.	Produces the shared 256-channel features supplied to the output branches.
Motion-output branches	The shared feature map is passed to three independent branches. Each branch contains a DSEW block (256→512 channels, stride 2), global average pooling, and a 512→1 fully connected layer.	Produces one scalar per branch: $x_{g}$, $z_{g}$, or $\psi_{g}$.
The complete network contains approximately 35.84 million trainable parameters.		

**Table 2:** Sequence-wise RMSE statistics of ground-view motion estimates against the reference motion over twelve real-vehicle sequences.

Evaluated Components	Metric	PEME [31]	Proposed (Online Estimate)	Proposed (Refined Estimate)
$v_{xg}$	Sequence-wise RMSE ^1^	0.3364 ± 0.0841	**0.2175** ± 0.0229	0.2195 ± 0.0227
$v_{zg}$		0.3845 ± 0.1263	**0.3197** ± 0.1488	0.3215 ± 0.1502
$\omega_{\psi g}$		13.1399 ± 3.0290	44.9615 ± 8.7971	**7.4374** ± 1.2891

^1^ Each entry reports the mean ± sample standard deviation of the 12 sequence-wise RMSEs. The unit of $v_{xg}$ and $v_{zg}$ is m/s, and the unit of $\omega_{\psi g}$ is °/s. The lower mean error is highlighted in bold.

**Table 3:** Comparison of target-camera orientation calibration errors over twelve real-vehicle sequences.

Experiment Data	Error	SensorX2car [4]			Original Method [21]			Proposed Method		
		**Yaw**	**Pitch**	**Roll**	**Yaw**	**Pitch**	**Roll**	**Yaw**	**Pitch**	**Roll**
Sequence 1	Absolute Error ^1^	3.7874	0.2753	**0.1300**	0.5980	0.1925	1.1551	**0.1120**	**0.1017**	1.1051
Sequence 2		**0.0012**	**0.3073**	1.2350	2.0771	0.4781	0.5850	0.2173	0.4861	**0.5733**
Sequence 3		6.6587	**0.0336**	0.4881	4.4238	0.3369	**0.0343**	**0.8984**	0.3286	0.0344
Sequence 4		5.7546	**0.3161**	**0.7221**	7.3366	0.5023	0.8817	**0.1518**	0.5008	0.8942
Sequence 5		0.2778	**0.2042**	**0.7239**	0.8495	0.4092	0.9556	**0.1837**	0.2712	0.8008
Sequence 6		2.5193	**0.1221**	**0.3150**	0.5655	0.3688	1.3472	**0.0484**	0.3145	1.4364
Sequence 7		3.3735	0.1838	**1.3842**	2.0511	0.1054	1.7010	**0.0489**	**0.0320**	1.9743
Sequence 8		1.7072	**0.0615**	**0.8222**	5.5283	0.1105	1.4166	**0.5481**	0.0988	1.4138
Sequence 9		0.4611	0.4343	**0.9028**	0.2172	0.0923	1.4577	**0.1234**	**0.0891**	1.4548
Sequence 10		1.6977	**0.1032**	**0.5371**	0.1987	0.1851	2.1116	**0.1628**	0.1796	2.1873
Sequence 11		1.0375	**0.1194**	**0.5331**	2.9579	0.4369	2.7283	**0.0522**	0.2680	2.0554
Sequence 12		0.8515	0.2125	**0.4963**	2.3938	0.1954	1.9165	**0.5139**	**0.1587**	1.8285
Mean		2.3440	**0.1978**	**0.6908**	2.4331	0.2844	1.3576	**0.2551**	0.2358	1.3132

^1^ The unit of all results is °. Yaw angle, pitch angle and roll angle correspond to *y*-axis, *x*-axis, and *z*-axis, respectively. The lower error is highlighted in bold.

**Table 4:** Variability in target-camera orientation calibration errors across ten initializations under the 5°/0.03 m perturbation setting.

Experiment Data	Original Method [21] ^1^			Proposed Method ^1^		
	**Yaw**	**Pitch**	**Roll**	**Yaw**	**Pitch**	**Roll**
Sequence 1	0.0399 [0.5321, 0.6706]	0.0003 [0.1918, 0.1929]	0.0033 [1.1509, 1.1626]	0.0262 [0.0764, 0.1639]	0.0117 [0.0777, 0.1153]	0.1118 [0.9450, 1.3176]
Sequence 2	0.2087 [1.7800, 2.4352]	0.0001 [0.4780, 0.4782]	0.0001 [0.5848, 0.5852]	0.0588 [0.1079, 0.3025]	0.0010 [0.4843, 0.4872]	0.0013 [0.5711, 0.5752]
Sequence 3	0.2020 [4.1534, 4.6898]	0.0001 [0.3368, 0.3371]	0.0001 [0.0342, 0.0343]	0.0520 [0.8358, 0.9870]	0.0014 [0.3264, 0.3305]	0.0018 [0.0321, 0.0379]
Sequence 4	0.2355 [7.0430, 7.7989]	0.0001 [0.5021, 0.5024]	0.0001 [0.8816, 0.8819]	0.0577 [0.0805, 0.2198]	0.0053 [0.4919, 0.5066]	0.0138 [0.8639, 0.9118]
Sequence 5	0.1557 [0.5422, 1.0949]	0.0004 [0.4086, 0.4099]	0.0033 [0.9510, 0.9607]	0.0598 [0.1131, 0.2478]	0.0056 [0.2626, 0.2802]	0.0419 [0.7262, 0.8679]
Sequence 6	0.1458 [0.4185, 0.9366]	0.0003 [0.3682, 0.3693]	0.0037 [1.3415, 1.3524]	0.0210 [0.0311, 0.1029]	0.0163 [0.2901, 0.3507]	0.1373 [1.1989, 1.6799]
Sequence 7	0.1845 [1.6955, 2.2817]	0.0016 [0.1031, 0.1078]	0.0137 [1.6864, 1.7288]	0.0155 [0.0290, 0.0741]	0.0066 [0.0240, 0.0445]	0.0790 [1.8658, 2.0819]
Sequence 8	0.1181 [5.3974, 5.6759]	<0.0001 [0.110532, 0.110541]	<0.0001 [1.416630, 1.416650]	0.0584 [0.4750, 0.6416]	0.0015 [0.0960, 0.1008]	0.0022 [1.4105, 1.4175]
Sequence 9	0.0501 [0.1483, 0.3026]	<0.0001 [0.092224, 0.092387]	<0.0001 [1.457660, 1.457740]	0.0568 [0.0562, 0.2121]	0.0084 [0.0751, 0.1014]	0.0095 [1.4424, 1.4722]
Sequence 10	0.0254 [0.1483, 0.2374]	0.0004 [0.1841, 0.1858]	0.0061 [2.1012, 2.1198]	0.0228 [0.1244, 0.2018]	0.0126 [0.1547, 0.1930]	0.1211 [1.9325, 2.3368]
Sequence 11	0.1864 [2.5412, 3.2533]	0.0002 [0.4366, 0.4370]	0.0025 [2.7237, 2.7317]	0.0391 [0.0097, 0.0993]	0.0040 [0.2610, 0.2742]	0.1109 [1.8599, 2.2301]
Sequence 12	0.1572 [2.1011, 2.6281]	0.0002 [0.1950, 0.1957]	0.0028 [1.9107, 1.9212]	0.0223 [0.4856, 0.5573]	0.0091 [0.1446, 0.1724]	0.1090 [1.6993, 2.0817]

^1^ Each cell reports the sample standard deviation on the first line and [minimum, maximum] on the second line, calculated from the ten trial-level absolute errors. Standard deviations below 0.0001° are reported as <0.0001, with six decimal places retained for the corresponding ranges. The unit of all results is °.

**Table 5:** Mean target-camera orientation calibration errors of the proposed method under different initial extrinsic perturbation levels.

Initial Extrinsic Perturbation		Mean Absolute Error ^1^		
**Rotation-Angle Magnitude (°)**	**Translation-Component Magnitude (m)**	**Yaw**	**Pitch**	**Roll**
2	0.01	0.2412	0.2367	1.3139
5	0.03	0.2551	0.2358	1.3132
10	0.05	0.2596	0.2361	1.3261
15	0.07	0.2717	0.2354	1.3198

^1^ The unit of the yaw, pitch, and roll errors is °.

## Data Availability

The datasets presented in this article are not readily available due to restrictions.
